# Lupus Flare: An Uncommon Presentation of Disseminated Gonorrhea

**DOI:** 10.1155/2014/626095

**Published:** 2014-06-15

**Authors:** Uyen To, Joyce Kim, David Chia

**Affiliations:** Yale School of Medicine, 333 Cedar Street, New Haven, CT 06510, USA

## Abstract

Gonorrhea is one of the most common sexually transmitted diseases in the US with 700,000 annual cases. Although most cases of gonorrhea are localized, approximately 0.5–3% become disseminated. Here we discuss a rare case of a patient with systemic lupus erythematosus (SLE) who developed septic shock from disseminated gonorrhea infection (DGI). Our patient is a 24-year-old woman with SLE, mixed connective tissue disease with cutaneous vasculitis, and lupus nephritis who presented with several weeks of malaise and generalized body aches associated with a diffuse rash along her fingers, palms, and trunk. Infectious workup was unrevealing with the exception of a positive gonorrhea test obtained from a cervical swab. Given her symptoms of tenosynovitis, the appearance of her skin lesions, and her positive gonorrhea test, she was diagnosed with septic shock secondary to DGI. With antibiotic treatment, the patient reported a dramatic improvement of the pain in her swollen joints and her rash receded. Patients diagnosed with SLE carry an increased risk of gonorrhea regardless of whether or not they are being treated for their SLE. Although it is well-documented that SLE is associated with severe DGI, few describe it resulting in overt septic shock.

## 1. Introduction

Gonorrhea is one of the most common sexually transmitted diseases in the US with 700,000 annual cases [1]. Epidemiologically, there has been a decline in overall prevalence since 1941; however, the emergence of drug-resistant gonorrhea has become a growing concern [2]. Although most cases of gonorrhea are localized to the mucosal surfaces of the body, approximately 0.5–3% become disseminated [1]. Populations with increased susceptibility and at higher risk of disseminated infection include patients with systemic lupus erythematosus (SLE) and complement deficiencies [3]. Here we discuss a rare case of a patient with SLE who developed septic shock from disseminated gonorrhea infection (DGI).

## 2. Case Report

Our patient is a 24-year-old woman with SLE, mixed connective tissue disease (MCTD) with cutaneous vasculitis, and lupus nephritis who presented with malaise and generalized body aches. She was in her usual state of health until three weeks prior to admission when she developed low grade fevers, night sweats, generalized fatigue, and arthralgias of her shoulders and knees, all of which she reported to be similar to her symptoms of MCTD. During the two weeks prior to admission, she endorsed a progressive rash along her fingers, palms, and trunk, which she described as similar to those she experienced from her cutaneous vasculitis. For her rheumatologic conditions, she had not required any recent treatments with corticosteroids or hydroxychloroquine. She denied any history of travel or recent sick contacts. Review of systems was notable for malodourous vaginal discharge but was otherwise unremarkable. She had no prior history of sexually transmitted diseases and tested negative for chlamydia and gonorrhea five months prior to presentation. Since then she has had two new sexual partners.

In the emergency department, she was observed to be febrile, tachycardic, and hypotensive. On physical exam, the patient was somnolent, fatigued, and ill-appearing. She had dry mucous membranes with no lymphadenopathy. Her lung and cardiac exams were normal. She had a palpable spleen, but no hepatomegaly or abdominal tenderness. Her skin was notable for multiple dark pustules with most approximating 1 cm × 1 cm along her arms, legs, palms, soles, and abdomen. There were linear excoriations around the lesions. Her wrists, knees, and ankles were swollen and tender to the touch. Range of motion was limited due to severe pain.

Resuscitation was initiated with intravenous crystalloid infusion, while blood and urine cultures were obtained and broad-spectrum antibiotics (vancomycin, levofloxacin, and metronidazole) started.

Subsequently, she was admitted to the MICU for septic shock after she exhibited persistent hypotension refractory to a total of 7 L of intravenous fluids. As a result, she was administered stress-dose steroids due to clinical concern for adrenal insufficiency and was started on a norepinephrine infusion. She underwent a CT scan of the chest, abdomen, and pelvis, RUQ ultrasound, echocardiogram, and pelvic exam. Her imaging studies failed to reveal an infectious source and her blood and urine cultures demonstrated no growth, but her cervical swab obtained from the pelvic exam was positive for gonorrhea and negative for chlamydia. Her complement levels were measured and were notable for low C3, C4, and total complement levels (40 mg/dL, 8 mg/dL, and <10 mg/dL, resp.). Given her symptoms of tenosynovitis, the appearance of her skin lesions, and her positive gonorrhea test, she was diagnosed with septic shock secondary to DGI.

Consequently, she was narrowed to ceftriaxone and was gradually weaned off pressors. With appropriate antibiotic treatment, the patient reported a dramatic improvement of the pain in her swollen joints and her rash receded. She was eventually transitioned to cefpodoxmine and discharged home to complete a full 10-day course of treatment.

## 3. Discussion

Disseminated gonorrhea infection can manifest itself in a variety of ways with symptoms commonly classified into the early or late stage [4]. Our patient exhibited early-stage signs with symptoms of bacteremia including high fevers and rigors with migratory and painful joints that can involve the knees, elbows, and distal joints [5]. Characteristic skin lesions are often another early finding and may occur in approximately 75% of patients. Although the appearance of the skin lesions varies, they are usually painless macules and papules that can evolve into pustules with a hemorrhagic component [6]. Other dermatologic manifestations include dermatitis, erythema nodosum, and erythema multiforme [5]. Late-stage symptoms include septic arthritis, endocarditis, perihepatitis, osteomyelitis, pericarditis, and meningitis [5, 7].

DGI should be considered and not overlooked, particularly in patients with SLE. Not only do the symptoms of these two disease processes have significant overlap, but also patients diagnosed with SLE carry an increased risk for severe gonorrheal infection due to the immunosuppressive state from being on steroids or other immunosuppressive agents, complement deficiencies, and an impaired reticuloendothelial system [3, 8]. The complement system is key to clearing* Neisseria gonorrhea* species, where the terminal complement complex is vital for its clearance by the immune system [9]. The terminal complex is involved in the lysis of the thick cell wall of encapsulated bacteria and thus its absence can lead to disseminated infection [9]. Testing for complement deficiency includes evaluating for CH50 and total complement, which are generally both low in these individuals [9]. Other risk factors for severe gonorrheal infections in the SLE population are young age, female gender, and concurrent renal disease, which are all characteristics present in our patient [10]. Though it is well-documented that SLE is associated with DGI, few describe it resulting in overt septic shock, like in this case [10].

Early identification of the disease process and initiation of appropriate antibiotics are essential to prevent late manifestations of the infection [5]. With the rapid emergence of fluoroquinolone-resistant strains of* N. gonorrhea*, it is now recommended that patients be hospitalized for DGI and treated with intravenous third generation cephalosporins. Preferred agents include ceftriaxone 1 g daily or cefotaxime/ceftizoxime 1 g every 8 hours [2]. These regimens should be continued for at least 24–48 hours before considering transition to oral antibiotics, namely, cefixime 400 mg every 12 hours or a fluoroquinolone (if susceptible). Antibiotic treatment should continue for a total of 7–10 days [5] but extended to a 14-day course in patients with SLE [10]. Those who are allergic to cephalosporins may be treated with intravenous erythromycin or streptomycin instead [5]. Standard care also includes treatment of recent sexual partners who may harbor and spread the infection otherwise [1].
